# Trauma care in Brazil: a comparative study of patient characteristics at public *versus* private hospitals

**DOI:** 10.1590/acb414026

**Published:** 2026-07-24

**Authors:** Brenda Feres, Sarah Lopes Salomão, Luiza Telles, Paulo Henrique Melo, Lauren Kratky, Jaqueline Dourado, Esther Louise Freire Costa, David Patrick Mooney, Gabriel Schnitman

**Affiliations:** 1 Kursk State Medical University – Kursk – Russia; 2 Universidade de São Paulo – Hospital for Rehabilitation of Craniofacial Anomalies – Bauru (SP) – Brazil; 3 Universidade de São Paulo – Faculdade de Medicina – São Paulo (SP) – Brazil; 4 McGill University – Department of Surgical and Interventional Sciences – Montreal – Canada; 5 Montreal Children’s Hospital – Harvey E. Beardmore Division of Pediatric Surgery – Montreal – Canada; 6 Harvard University – Medical School – Program in Global Surgery and Social Change – Boston (MA) – United States.; 7 Dartmouth-Hitchcock Medical Center – Department of Surgery – Lebanon (NH) – United States.; 8 Escola Bahiana de Medicina e Saúde Pública – Faculdade de Medicina – Salvador (BA) – Brazil; 9 Universidade Federal da Bahia – Faculdade de Medicina – Salvador (BA) – Brazil; 10 Boston Children’s Hospital – Pediatric Surgery – Boston (MA) – United States.

**Keywords:** Wounds and Injuries, Hospitals, Public, Hospitals, Private, Brazil

## Abstract

**Purpose:**

Trauma remains a leading cause of hospital admissions worldwide, particularly in low- and middle-income countries. Brazil’s dual healthcare system reflects disparities in resource availability and clinical outcomes. This study compared epidemiological profiles of trauma patients treated at a public *versus* a private hospital in Salvador, Bahia, Brazil.

**Methods:**

We retrospectively analyzed 6,472 trauma-related hospitalizations from August 2021 to August 2023. Data were extracted from Hospital Information System/Department of Informatics of the Unified Health System and a private hospital database. Patients admitted under the 10th revision of the International Classification of Diseases, categories S00–T88, were included, excluding burns and orthopedic injuries. A χ^2^ test assessed associations between trauma mechanisms and hospitals.

**Results:**

Among the 6,472 cases, 6,342 occurred in the public hospital and 130 in the private hospital. In the public hospital, males accounted for 82.1% of the cases, and the leading causes were motorcycle crashes (21.7%) and gunshot wounds (16.2%). At the private hospital 58.4% of patients were males, with falls (50.8%) predominating among older adults. Violence-related injuries predominated in the public hospital, while falls predominated in the private hospital.

**Conclusion:**

This study revealed distinct trauma patterns in the public *versus* the private hospital in one Brazilian city. The public hospital treated younger male victims of violence, while the private hospital treated older patients with falls. These findings underscore the need for policies addressing trauma care disparities.

## Introduction

Trauma is the leading cause of death worldwide, impacting public health and the global economy. The World Health Organization reports that approximately five million deaths annually can be attributed to trauma, primarily due to road traffic fatalities and interpersonal violence^1,2^. This burden is especially severe in low- and middle-income countries^3^, where nearly 90% of road traffic fatalities occur, despite these countries having only 60% of the world’s vehicles^4^. Trauma remains a leading cause of death in Brazil and places a substantial burden on both public and private healthcare systems. Data from the Brazilian Ministry of Health consistently ranks trauma among the top five causes of death, with road traffic fatalities, assaults, and falls being the most common ones^5^. Despite public health efforts in Brazil that have reduced road traffic fatalities, the overall burden remains high, resulting in frequent hospitalizations and long-term disabilities^6^.

Brazil’s healthcare system is divided into two main sectors: the public system, represented by the Unified Health System (SUS), and the private sector, composed of privately funded hospitals and health insurance providers. These two sectors’ differences go beyond funding and administration and extend to the availability of resources, staffing, and overall quality of care. Public hospitals under the SUS serve a vast segment of the population and are critical to providing trauma care. However, they often contend with challenges such as overcrowding, supply shortages, and limited staffing. In contrast, private hospitals, which primarily serve insured patients or those who can pay out of pocket, generally have greater access to resources, advanced technology, and shorter wait times, all of which can influence outcomes in trauma patients^7^.

Previous studies suggested that the type of hospital a patient presents to can have a significant impact on clinical outcomes in trauma. In countries such as the United States of America, there is evidence that patients treated in level 1 trauma centers, which are generally well-equipped and have specialized trauma teams, have lower mortality rates than those treated in hospitals that are not trauma centers^8^. In Brazil, where there is a clear distinction between public and private hospitals, these differences may be even more pronounced. There are very few studies on this topic, and those that exist are often limited by small sample size or focus on a specific region in Brazil, limiting generalizability^9^.

Comparing the epidemiologic profiles of trauma patients between public and private hospitals in Brazil is crucial to identifying possible disparities in access and quality of care. These disparities, if present, can guide public policies aimed at equity in emergency care and allow a more efficient allocation of resources. Understanding these disparities is also essential for designing strategies to improve clinical outcomes and reduce trauma mortality throughout Brazil.

This study aimed to compare the epidemiological profiles of trauma patients treated in a public hospital and a private hospital in Salvador, Bahia, Brazil. By analyzing patient demographics, trauma mechanisms, and clinical outcomes, it aimed to provide a comprehensive overview of potential differences in trauma care between public and private sectors. These findings may inform strategies to enhance equity in trauma care across Brazil.

## Methods

### Study design

This retrospective study compared trauma patients from August 2021 to August 2023 in two hospitals in Salvador, Bahia, Brazil. One hospital is a private hospital (Hospital Santa Izabel), and the other hospital is a public hospital (Hospital Geral do Estado).

To contextualize, Salvador, the capital of Bahia, exhibits a unique economic profile in Brazil and the Northeast region. Despite having the lowest gross domestic product *per capita* among Brazilian capitals, at R$ 20,417.14 (approximately US$ 3,500), Salvador remains the largest economy in the Northeast^10^. This gain underscores Salvador’s economic potential despite broader regional disparities, highlighting the role of targeted public and private investments in fostering urban economic development. However, this fact contrasts with the number of hospitalizations due to trauma in Salvador, such as traffic accidents, burns, and assaults. The capital had a total of 26,794 trauma-related hospitalizations in 2023, which is 63% higher than the national average. These data reinforce the importance of the issue portrayed in this study^11^.

### Data sources for public hospitals

We used data from the Brazilian Hospital Information System (SIH) database, part of the Department of Informatics of the Unified Health System (DATASUS). DATASUS includes data from all public hospitals across Brazil, encompassing patient demographics, hospital admissions, procedures performed, as well as diagnoses. The extraction and initial processing of the data were performed using R Studio version 2022.07, by the ‘microdatasus’ package^12^. We included all hospitalizations that had trauma-related diagnoses coded as primary or secondary diagnoses according to the International Classification of Diseases, 10th revision (ICD-10). Specifically, we used ICD-10 code intervals S00-T88, which encompass a wide range of injuries and trauma-related conditions. The S00–S99 codes cover injuries to specific body regions—head (S00–S09), neck (S10–S19), thorax (S20–S29), abdomen, and pelvis (S30–S39), and limbs (S40–S99)—and include fractures, dislocations, open wounds, and superficial injuries. The T00–T88 codes include injuries involving multiple body regions (T00–T07). Patients with burns and orthopedic trauma injuries were excluded, as they are often referred to plastic and orthopedic surgery departments, instead of being managed by trauma surgeons.

### Data sources for private hospitals

The data from the private hospital, Santa Izabel, was obtained from the hospital’s internal database, which systematically records all trauma cases treated within its facilities. All trauma mechanisms are identified in Table 1. This database includes detailed information, such as demographics, mechanism of injury, hospital length of stay, and outcomes, allowing for comprehensive retrospective data analysis. To maintain patient privacy, only de-identified information was used.

**Table 1:** General data by mechanism of trauma, in the private hospital and the public hospital.

	Public hospital					Private hospital			
	NOC n, (%)	Age	LOS	NOD		NOC n, (%)	Age	LOS	NOD
Total	6,342	35.4 (1–98)	7.2	129		130	58.3 (10–101)	8.4	2
Diving in shallow water	3 (0.05)	33.67 (17–45)	12.67	0		1 (0.77)	67	76.00	0
Gunshot wound	1,030 (16.24)	29.98 (1–89)	7.61	34		12 (9.23)	48.92 (26–63)	13.75	0
Motorcycle crashes	1,376 (21.70)	32.72 (2–87)	7.63	22		18 (13.85)	30.06 (15–65)	12.06	0
Motor vehicle crashes	154 (2.43)	35.75 (2–75)	8.77	2		14 (10.77)	48.43 (23–68)	8.00	0
Fall from a height	699 (11.02)	44.38 (1–92)	9.20	27		33 (25.38)	65.82 (10–94)	7.12	1
Ground-level fall	437 (6.89)	51,20 (1–98)	10.13	21		33 (25.38)	76.42 (43–101)	6.82	1
Stab wound	773 (12.19)	32,83 (1–79)	4.97	5		3 (2.31)	39.33 (32–51)	6.00	0
Crush injury	73 (1.15)	27,03 (1–68)	4.03	1		1 (0.77)	33	4.00	0
Kite surfing accident	0 (0)	0	0	0		1 (0.77)	61	4.00	0
Bicycle crash	0 (0)	0	0	0		4 (3.08)	51 (46–57)	3.50	0
Self-harm	0 (0)	0	0	0		2 (1.54)	54.50 (50–59)	3.50	0
Pedestrian trauma	339 (5.35)	40.32 (1–87)	9.74	7		6 (4.62)	72.83 (18–83)	3.17	0
Physical aggression	645 (10.17)	34.08 (1–89)	7.21	4		2 (1.54)	29.50 (18–41)	1.50	0
Airway obstruction by foreign body	3 (0.05)	29.33 (11–57)	1.00	0		0 (0)	0	0	0
Blunt object	3 (0.05)	11.33 (7–19)	4.00	0		0 (0)	0	0	0
Drowning	3 (0.05)	14.33 (3–37)	9.33	0		0 (0)	0	0	0
Explosion	76 (1.2)	34.74 (2–81)	4.93	4		0 (0)	0	0	0
Machinery	296 (4.7)	42.62 (0–83)	4.76	0		0 (0)	0	0	0
Natural phenomena	35 (0.6)	32.09 (11–57)	4.91	0		0 (0)	0	0	0
Non-powered hand tools	2 (0.03)	23 (4–42)	8.00	0		0 (0)	0	0	0
Penetrating eye or natural orifice injury	325 (5.1)	24.43 (1–87)	2.16	2		0 (0)	0	0	0
Skin laceration or penetration	54 (0.85)	32.28 (1–97)	4.44	0		0 (0)	0	0	0
Unspecified	16 (0.25)	31.81 (2–57)	2.75	0		0 (0)	0	0	0

NOC: number of cases; Age: mean age (range) in years; LOS: length of stay in days; NOD: number of deaths. Source: Elaborated by the authors.

### Data analysis

Descriptive statistics were calculated to summarize patient demographics, length of hospital stay, and outcomes. Continuous variables were expressed as means and standard deviations or medians and interquartile ranges, depending on data distribution. A χ^2^ test was performed to assess the association between the mechanism of trauma and hospital type. Expected frequencies were checked, and Fisher’s exact test was used when any cell had an expected count < 5.

### Ethical considerations

This study utilized de-identified, publicly available data from the SIH/SUS database. According to Brazilian National Health Council Resolution no. 466/2012, research using public domain data without individual identifiers does not require approval from an ethics committee.

## Results

From 2021 to 2023, a total of 6,472 trauma cases were recorded. Among them, 6,342 trauma patients were seen at the public hospital, while 130 cases were seen at the private hospital (Table 1). The most frequent trauma mechanisms at the public hospital were motorcycle crashes (21.7%), gunshot wounds (16.2%), and stab wounds (12.2%), with an average patient age by mechanism of 32.7, 30, and 32.8 years old, respectively. In the private hospital, most of the trauma mechanisms were falls, with both falls from a height and ground-level falls each representing 25.4% of the total cases, followed by motorcycle crashes representing 13.9%, with an average patient age by mechanism of 65.8, 78.4, and 30.1 years old, respectively. Stab wounds and physical aggression were less frequent in the private hospital, 2.3 and 1.5%, respectively. These patterns highlight a higher prevalence of violence and motorcycle-related injuries in the public hospital and a predominance of geriatric falls in the private hospital. A χ^2^ analysis revealed a statistically significant difference in the distribution of trauma mechanisms between public and private hospitals (χ^2^ = 513.9, degree of freedom [df] = 22, *p* < 0.001).

Certain injury mechanisms in both public and private hospitals exhibited gender disparities. Females accounted for only 12.3% of patients with gunshot wounds in the public hospital and 8.3% in the private hospital. Females also only represented 10.8% of motorcycle crash victims in the public hospital and 16.7% in the private hospital. In contrast, motor vehicle crashes and falls from a height in the private hospital exhibited less gender disparity, with women representing 50 and 45.5% of cases, respectively. The least common trauma mechanisms in both hospitals included blunt object, drowning, and airway obstruction by foreign bodies, each representing fewer than 0.1% of total cases, with an equal gender distribution in many of these rare instances.

### Demographics of patients by trauma mechanisms and hospital type

In both hospitals, age distribution varied significantly across trauma mechanisms. Patients involved in motorcycle crashes tended to be younger, with an average age of 32.7 years old at the public hospital and 30.1 at the private hospital. Gunshot wounds, particularly in the public hospital, also involved younger patients, with an average age of 30. In contrast, falls from standing height predominantly affected older adults, with an average age of 44.4 years old in the public hospital and 65.8 years old in the private hospital, underscoring the higher prevalence of geriatric trauma in private care.

Gender distribution also showed distinct patterns by trauma mechanism. Males were overwhelmingly represented in most trauma categories. In the public hospital, 82.1% of all traumas involved male patients. This proportion is more balanced in the private hospital, where 58.5% of the cases were male patients. Particularly in motorcycle crashes, 89.2% of cases in the public hospital and 83.3% in the private hospital were male. Gunshot wounds also presented a marked difference: the male population represented 87.7% of cases in the public hospital and 91.7% in the private hospital (Table 2).

**Table 2:** Mechanisms of trauma, by gender, in the public hospital and the private hospital.

	Public hospital				Private hospital		
	Female n (%)	Male n (%)	Total (n)		Female n (%)	Male n (%)	Total (n)
Total	1,134 (17.88)	5,208 (82.12)	6,342		54 (41.54)	76 (58.46)	130
Diving in shallow water	0 (0)	3 (100)	3		0 (0)	1 (100)	1
Gunshot wound	127 (12.33)	903 (87.67)	1,030		1 (8.33)	11 (91.67)	12
Motorcycle crashes	149 (10.83)	1227 (89.17)	1,376		3 (16.67)	15 (83.33)	18
Motor vehicle crashes	29 (18.83)	125 (81.17)	154		7 (50)	7 (50)	14
Fall from a height	156 (22.32)	543 (77.68)	699		15 (45.45)	18 (54.55)	33
Ground-level fall	140 (32.04)	297 (67.96)	437		21 (63.64)	12 (36.36)	33
Stab wound	180 (23.29)	593 (76.71)	773		0 (0)	3 (100)	3
Crush injury	16 (21.92)	57 (78.08)	73		1 (100)	0 (0)	1
Kite surfing accident	0 (0)	0 (0)	0		0 (0)	1 (100)	1
Bicycle crash	0 (0)	0 (0)	0		0 (0)	4 (100)	4
Self-harm	0 (0)	0 (0)	0		2 (100)	0 (0)	2
Pedestrian trauma	81 (23.89)	258 (76.11)	339		4 (66.67)	2 (33.33)	6
Physical aggression	91 (14.11)	554 (85.89)	645		0 (0)	2 (100)	2
Airway obstruction by foreign body	1 (33.33)	2 (66.67)	3		0 (0)	0 (0)	0
Blunt object	0 (0)	3 (100)	3		0 (0)	0 (0)	0
Drowning	2 (66.67)	1 (33.33)	3		0 (0)	0 (0)	0
Explosion	14 (18.42)	62 (81.58)	76		0 (0)	0 (0)	0
Machinery	15 (5.07)	281 (94.93)	296		0 (0)	0 (0)	0
Natural phenomena	10 (28.57)	25 (71.43)	35		0 (0)	0 (0)	0
Non-powered hand tools	1 (50)	1 (50)	2		0 (0)	0 (0)	0
Penetrating eye or natural orifice injury	103 (31.69)	222 (68.31)	325		0 (0)	0 (0)	0
Skin laceration or penetration	17 (31.48)	37 (68.52)	54		0 (0)	0 (0)	0
Unspecified	2 (12.50)	14 (87.50)	16		0 (0)	0 (0)	0

Source: Elaborated by the authors.

### Length of stay by trauma mechanisms and hospital type

The average hospital length of stay differed between the two institutions for gunshot wounds (7.6 days in the public hospital *versus* 13.8 days in the private hospital) and motorcycle crashes (7.6 days in the public hospital *versus* 12.1 days in the private hospital). A comparison of trauma mechanisms associated with the longest hospital stays suggests different patterns between the two hospitals. In the public hospital, diving in shallow water was associated with the longest mean length of stay (12.7 days; n = 3; mean age = 33.7 years old). In the private hospital, a single case of this mechanism was observed, with a length of stay of 76 days (age = 67 years old).

Among trauma cases, the public hospital reported 129 deaths (2%), compared to two (1.5%) at the private hospital. Gunshot wounds (26.3%), falls from height (20.9%), and motorcycle crashes (17.1%) were the leading causes of death in the public hospital. In contrast, the only fatalities at the private hospital resulted from falls.

## Discussion

Trauma is the leading cause of mortality among Brazilian youth, with men being particularly affected. This study reveals differences in trauma patterns and outcomes between two hospitals: one public and one private, in the same city in Brazil. In the public hospital, motorcycle crashes and gunshot wounds were the leading causes of mortality, reflecting socioeconomic and occupational risks. Meanwhile, in the private hospital, there was a more balanced gender distribution, with ground-level falls as the most common cause, particularly among women, indicating vulnerabilities related to specific health conditions.

Gender disparities in trauma incidence were more pronounced in the public hospital, reflecting broader occupational and socioeconomic dynamics. At the public hospital, males accounted for the overwhelming most trauma cases. This aligns with Brazil’s Health Ministry information, indicating that men are more likely to be involved in traffic accidents^13^. It is important to highlight that motorcycles are often used by blue-collar workers for transportation or work, such as food delivery and motorcycle taxis. In Brazil, motorcyclists are predominantly male, especially in transportation and work-related groups^14,15^. In contrast, there was a more balanced gender representation in the private hospital. White-collar workers are more likely to seek care at private hospitals, where injuries commonly associated with occupational exposure, such as traffic accidents or machinery-related incidents, are less frequent^16^.

In the private hospital, there was a higher prevalence of injuries resulting from falls, representing more than 50% of all causes, particularly among older adults. In this hospital, ground-level falls had a mean age of 76.4 years old, while falls from height occurred in patients with a mean age of 65.8. This pattern suggests that trauma cases in the private hospital are less influenced by occupational hazards and more by age-related vulnerabilities. Moreover, there was a noticeable tendency for older patients to be involved in ground-level falls compared to falls from a height in both hospitals. The mean age for ground-level falls was 51.2 years old in the public hospital *versus* 44.4 for falls from a height. The private hospital’s mean age for ground-level falls was 76.4 *versus* 65.8 years old for falls from a height. This aligns with existing literature, which highlights that ground-level falls are particularly common among the elderly^17^.

Interestingly, ground-level falls in the private hospital had a higher female representation (63.6%), whereas in the public hospital, males were predominant (68%). This is most likely due to the older age profile of ground-level fall patients in the private hospital and may be attributed to the higher prevalence of osteoporosis among elderly women^18^. Osteoporosis significantly increases the risk of femoral fractures in women and increases the chances of having a fracture after a fall, further emphasizing how underlying health conditions shape trauma patterns in this demographic^19^.

As for violence-related injuries, male patients had a clear predominance in both hospitals, reflecting the influence of cultural aspects and traditional gender roles. Research on violence-related injuries in Brazil consistently shows a significant gender disparity, with males being disproportionately affected. Studies revealed that men are more likely to be victims of assault, while women are more prone to self-inflicted injuries^20^. The male-to-female ratio for violence-related incidents is particularly high, with mortality rates 11.6 times higher for men, hospitalization rates 4.5 times higher, and emergency treatment rates 2.8 times higher for men than women. Young men aged 15–29 are especially vulnerable, with mortality rates as high as 92.8 per 100,000 inhabitants^21^. Alcohol consumption is frequently associated with male victims of violence^20,22^. These gender disparities are attributed to cultural models and socio-structural factors, highlighting the need for targeted prevention strategies and further research into the underlying causes of violence in Brazil.

These disparities underscore the influence of the healthcare system structure on trauma outcomes. Private hospitals cater to a smaller, more selective patient population, allowing for individualized and intensive care, while public hospitals often manage a higher and more diverse caseload, which may explain the reason for poorer outcomes in certain metrics. These patterns highlight the need for strategic investments in public hospital systems to improve trauma care quality while addressing the structural differences that contribute to such divergent outcomes.

### Limitations

This study had some significant limitations. First, public and private hospitals record the mechanisms of trauma differently. The public database used ICD-10 codes, whereas the private hospital documented trauma mechanisms in free text. Another limitation was the smaller number of cases recorded in the private hospital compared to the public hospital, resulting in sparse data from the private hospital, especially when analyzing subgroups. Consequently, only two deaths were recorded in the private hospital, both related to falls, which prevented us from performing statistical comparisons involving death rates in the private hospital.

## Conclusion

This study revealed distinct epidemiological trauma patterns in public *versus* private hospitals in Brazil. The public hospital predominantly serves younger male victims of violence-related injuries, while the private hospital treats older patients with age-related trauma such as falls. The observed disparities in mortality rates, hospital length of stay, and access to public *versus* private hospital systems highlight the importance of targeted changes in public trauma care, especially in policy creation related to resource allocation. These findings can inform future research and policy initiatives focused on improving trauma care and reducing unnecessary fatalities in vulnerable populations.

## Data Availability

The data will be available upon request.
